# Identification of genetic variants in five chinese families with keratoconus: Pathogenicity analysis and characteristics of parental corneal topography

**DOI:** 10.3389/fgene.2022.978684

**Published:** 2022-10-06

**Authors:** Wan-Yu Cheng, Shang-Ying Yang, Xiao-Yu Huang, Fei-Yin Zi, Hui-Ping Li, Xun-Lun Sheng

**Affiliations:** ^1^ Ningxia Eye Hospital, People’s Hospital of Ningxia Hui Autonomous Region, Third Clinical Medical College of Ningxia Medical University, Yinchuan, China; ^2^ Gansu Aier Ophthalmology and Optometry Hospital, Lanzhou City, China

**Keywords:** keratoconus, genetic, variants, corneal topography, hereditary

## Abstract

**Purpose:** The study aims to identify genetic variants in five Chinese families with Keratoconus (KC) and describe the characteristics of parental corneal topography.

**Methods:** Fifteen participants, including five probands and ten parents from five Chinese families with KC, were recruited for genetic and clinical analyses. Targeted next-generation sequencing using a custom-designed panel for KC was applied on the probands for variant identification. Sanger sequencing and cosegregation analysis of the suspected pathogenic variants were performed on the family members. The pathogenicities of variants were evaluated according to the American College of Medical Genetics and Genomics guidelines (ACMG). Pentacam 3D anterior segment analysis system was applied for keratectasia detection and the Corvis ST for corneal biomechanics measurement. Fifteen parameters were recorded, including nine keratectasia indicators (BAD-D, TP, Kmax, Df, Db, Dp, Dt, Da, ARTH), six corneal biomechanical indicators (CBI, DA ratio, SP-A1, IR, bIOP, TBI).

**Results:** A total of six novel variants, including five missense variants and one frameshift variant, were detected in the *HMX1, SLC4A11, TGFBI, PIKFYVE,* and *ZEB1* genes in five probands, all of which showed co-segregation of genotype and clinical phenotype and were determined to be pathogenic. The genetic model was autosomal dominant (AD) in four families and autosomal recessive (AR) in 1 family. The analysis of keratectasia and corneal biomechanical indicators of the proband’s parents (first-generation relatives) in AD families revealed that there were several abnormal indexes in BAD-D, TP, Kmax, Df, Db, Dp, Dt, Da, CBI, DA ratio, SP-A1, IR, bIOP and TBI test indexes, showing clinical characteristics of incipient KC.

**Conclusion:** Our study shows that variants in *HMX1, SLC4A11, TGFBI, PIKFYVE,* and *ZEB1* were associated with KC. Our study extends the gene spectrum associated with KC, provides novel insights into KC phenotypic assessments, and contributes to early diagnosis for these patients.

## 1 Introduction

Keratoconus (KC) is a genetic eye disease characterized by an ectasia central or paracentral cornea that thins and tapers forward, often resulting in high levels of irregular astigmatism and significant loss of vision in advanced stages of blindness. The prevalence of KC ranges from 0.05 to 0.23% worldwide (Wei-Yun, 2018). KC occurs in all races, without gender differences, most often at puberty, and is currently the leading cause of corneal transplantation in developed countries (Himal et al., 2021). The pathogenesis of KC is still unclear, and it is generally believed that multiple factors (such as genetic factors, environmental factors, viral infections, auto-metabolism, and immunity) cause abnormal synthesis or degradation of corneal collagen cells (Bykhovskaya and Rabinowitz, 2020). With the development of genetics and genetic testing techniques, there is increasing evidence that genetics is an important factor in the pathogenesis of KC. Foreign findings show that 13.5% of KC patients have a family history (Matthew and Charles, 2001), and some of them can develop the disease even for two to three consecutive generations (Nowak and Marzena, 2011). The prevalence of relatives of KC patients is as high as 3.34% (Wang et al., 2010) to 25.3% (Steele et al., 2010). The study of the heritability of corneal traits in KC patients has made great progress with the continuous improvement of corneal morphological examination, and quantitative parameters such as corneal topography can also provide an objective reference for the early diagnosis of KC (Steele et al., 2010). Wang et al. (Wang et al., 2010), by performing corneal topography on the first-degree KC relatives, found that the prevalence was 15–67 times higher in the first-degree relatives than in the general population. Genetic studies of genes related to KC using pathogenic gene variant analysis showed that the occurrence of KC has obvious genetic heterogeneity, and many genes in the nuclear genome such as *VSX1, ZEB1, LOX,* and *SOD1* have important roles in the occurrence of such disease.

The early stage of KC is more challenging to diagnose accurately due to the lack of typical clinical signs. Pentacam 3D anterior segment analysis system, Belin analysis system, and biomechanical analysis software can accurately capture corneal morphological and mechanical indices. The combined indices are more sensitive and help detect early corneal morphological abnormalities (Steele et al., 2010; Li et al., 2020). Target region sequencing combined with high-throughput sequencing technology (panel), in which specific gene capture probes capture multiple target region DNA fragments of related genes. Then, the captured target region DNA sequences are measured using second-generation sequencing technology to identify the pathogenic genes and potentially pathogenic variants (Xie et al., 2015). The advantage is the ability to achieve deeper sequencing depth and coverage in protein-coding regions, implying high accuracy and allowing for disease-focused, high-performance testing. This study performed corneal topography and corneal biomechanics in KC probands and their first-generation relatives to obtain keratectasia and corneal biomechanics-related indexes. Target region sequencing combined with high-throughput sequencing technology was used to screen relevant gene variants. Bioinformatics analysis results obtained candidate pathogenic variants. Sanger sequencing was used for validation and family co-segregation analysis to identify pathogenic variants. The pathogenic variants were analyzed in patients and parental phenotypes.

## 2 Materials and methods

### 2.1 Diagnostic criteria for Keratoconus

The study protocol was approved and reviewed by the Ethics Committee on Human Research at People Hospital of Ningxia Hui Autonomous Region. Written informed consent was received from each participant or their legal guardians before participation, and the study adhered to the tenets of the declaration of Helsinki.

The clinical diagnostic criteria of KC include 1) progressive myopia, irregular astigmatism, and poor correction with frame glasses; 2) typical changes such as abnormal elevation of the anterior and posterior surfaces of KC found on corneal topography; central or paracentral corneal thinning; 3) corneal biomechanical examination showing decreased biomechanical indexes such as corneal hysteresis and corneal resistance factor. This study used the diagnostic criteria and staging and grading criteria for KC from the Chinese Expert Consensus on the Diagnosis and Treatment of Keratoconus (2019) (Chinese Medical Association Ophthalmology Branch Corneal Disease Group, 2019).

Staging criteria: 1) Latent stage: Contralateral eye with a confirmed KC in one eye, having typical corneal topography and normal vision with uncorrected visual acuity (UCVA) ≥ 1.0; 2) Incipient stage: A confirmed diagnosis as KC with the best spectacle-corrected visual acuity (BSCVA) ≥ 0.8; 3) Completion stage: A confirmed diagnosis as KC with BSCVA <0.8, accompanied by the typical clinical signs of KC, classified into three levels; 4) Scar stage: It refers specifically to the residual scarring of the whole cornea after the acute KC edema has subsided.

Grading criteria for completion period: Grade 1: Corneal curvature <53.0 D, corneal thickness at the thinnest point >400 μm, BSCVA <0.8; Grade 2: Corneal curvature <55.0 D, corneal thickness at the thinnest point >300 μm, BSCVA<0.3; Grade 3: Corneal curvature >55.0 D, corneal thickness at the thinnest point ≤300 μm, BSCVA<0.05.

### 2.2 Clinical data collection

Clinical data were collected from KC probands and parents (the first generation of relatives), and detailed medical and family histories were asked. All standard ophthalmic examinations of participants were performed by comprehensive refractometry (VT-10, TOPCON, Japan), slit-lamp biomicroscopy (Topcon, Tokyo, Japan), intraocular pressure (IOP), anterior segment analysis system (Pentacam 70,700, Germany), and corneal biomechanics analyzer (Corvis ST 72100, Germany). Slit lamp biomicroscopy was used to identify stromal corneal thinning, Vogt’s striae, or a Fleischer ring. Best spectacle-corrected visual acuity (BSCVA) was performed by comprehensive optometry.

Anterior segment analysis system measured nine corneal parameters of the thinnest point thickness (TP), max keratometry (Kmax), Belin/Ambrósio enhanced ectasia display final D value (BAD-D), deviation of normality of the front elevation (Df), deviation of normality of the back elevation (Db), deviation of normality of pachymetric progression (Dp), deviation of normality of corneal thinnest point (Dt), deviation of normality of relational thickness (Da), and Ambrósio’s relational thickness (ARTH). And six other corneal parameters of corvis biomechanical index (CBI), deformation amplitude ratio (Da ratios), adjusted AP1-bIOP (SP-A1), biomechanical intraocular pressure (bIOP), integrated radius, and tomographic and biomechanical index (TBI) were recorded by corneal biomechanics analyzer. All results of corneal parameters will generate the corresponding quality factor (QS). When QS>95%, an “OK” display appears on the test instrument, indicating that the test data quality is acceptable. If the test quality is not good enough, it must be retested. To avoid detection errors, the operations mentioned above should be effectively checked by the same experienced medical technician at least three times. The higher quality results will be selected for the test group.

Evaluation of abnormal indicators: as studied by Ambrósio et al. (2011) (Garcia-Castellanos et al., 2017)^,^ normal values (white) were <1.6 SD (standard deviation), suspicious values (yellow) were 1.6–2.6 SD, and pathological values (red) > 2.6 SD for Df/Db/Dt/Dp/Da; Suspicious value (yellow) ranging between 1.6-3 SD and pathological values (red) > 3 SD for D value were used as a reference for index threshold. Suspicious value (yellow) ranging between 0.25–0.5 and pathological value (red) > 0.5 for CBI was used as a reference for the index threshold; Suspicious value (yellow) ranging between 0.25–0.75 and the pathological value (red) > 0.75 for TBI was used as a reference for index threshold.

### 2.3 Whole genome deoxyribonucleic acid extraction and sequencing analysis

5 ml of peripheral venous blood was drawn from each of the included family members, and genomic DNA was extracted. The Gen cap liquid phase capture kit jointly developed with MyGenostics, Beijing, China, was used to detect the exon regions, adjacent intron regions (50 bp), and known intron variants of 111 hereditary keratopathy-related genes. Sequencing was performed on Next-generation sequencing (NGS) technology (Illumina). Align and identify genetic variants with the UCSC hg19 human reference genome sequence using section. Sanger validation and family co-segregation analysis were performed for suspected pathogenic variants. The mutations were verified by Sanger sequencing and Q-PCR (quantitative fluorescence PCR). *TGFBI* gene, Q-PCR primer sequence: Forward primer F946-B9_F: 5 ′-GTA​ACT​GTG​AAC​TGT​GCC​CG-3'. Reverse primer R913-E9_R: 3 ′-GGA​GCC​CTT​TCT​CCC​TGA​G -5 '. SLC4A11 gene, Q-PCR primer sequence: Forward primer F913-E9_F: 5′- CAG​TCG​GAC​AGG​ATC​TCT​CG -3'. Reverse primer R913-E9_R: 3 ′- GGA​GCC​CTT​TCT​CCC​TGA​G -5 '. *PIKFYVE* gene, Q-PCR primer sequence: Forward primer F913-C5_F: 5′-CGG​TAC​TGC​TAC​CAG​GAT​TTG-3'. Reverse primer R913-C5_R: 3 ′-AAC​AGA​AAG​CAA​GGC​ATG​AAT​C -5 '.

### 2.4 In silico analysis

Database tools such as HGMD (human gene mutation database) and dbSNP (https://www.ncbi.nlm. nih.gov/snp/) were used to query the target mutation, check if they were reported pathogenic mutations in HGMD, and see if they were included. In the case of novel variants that have not been reported, the pathogenicity of the *de novo* variants was assessed for genetic variation according to the Standards and Guidelines for Interpretation of Sequence Variants published by the American College of Medical Genetics and Genomics (ACMG) in 2015. The variant sites were filtered and screened by putting them into the normal human database, including the normal population gene frequency 1,000 genomes (1,000 genomes), ESP6500 (NHLBI Exome Sequencing Project), EXAC (The Exome Aggregation Consortium), and EXAC-EAS (about 4,000 East Asians data under EXAC). The effects of mutation sites on protein function were predicted at SIFT (http://sift.jcvi.Org/www/SIFT_chr_coords_submit.html), Polyphen-2 (http://genetics.bwh.harvard.edu/pph2/) and Mutation Taster (http://mutationtaster.org/). Sites such as GERP++ (https://bio.tools/gerp) were used to measure the conservation of gene sequences across species in evolution. The predicted score of PolyPhen-2 (HDIV/HVAR) is close to 1, indicating that it is probably damaging (D); otherwise, it is possibly damaging (P) or benign (B). They were analyzed by SIFT, and amino acids with probabilities <0.05 are predicted deleterious (D). The scores of Mutation Taster analysis are between 0 and 1; disease probability is higher as the score nears. As for the range of predicted values of amino acid sequence conservativeness, GERP++, greater than 2, indicates relatively conservative. A high GERP score implies that the sequence is highly conserved and deleterious. Finally, the pathogenicity of the variants was assessed according to the standards and guidelines published in 2015 by the American College of Medical Genetics and Genomics (ACMG). The spatial structure of these wild type and mutated protein were modeled by Alpha Fold 2 and Misssense 3D, then were aligned with PyMOL 2.3 software.

## 3 Results

### 3.1 Clinical phenotype and genetic test results

**FIGURE 1:**
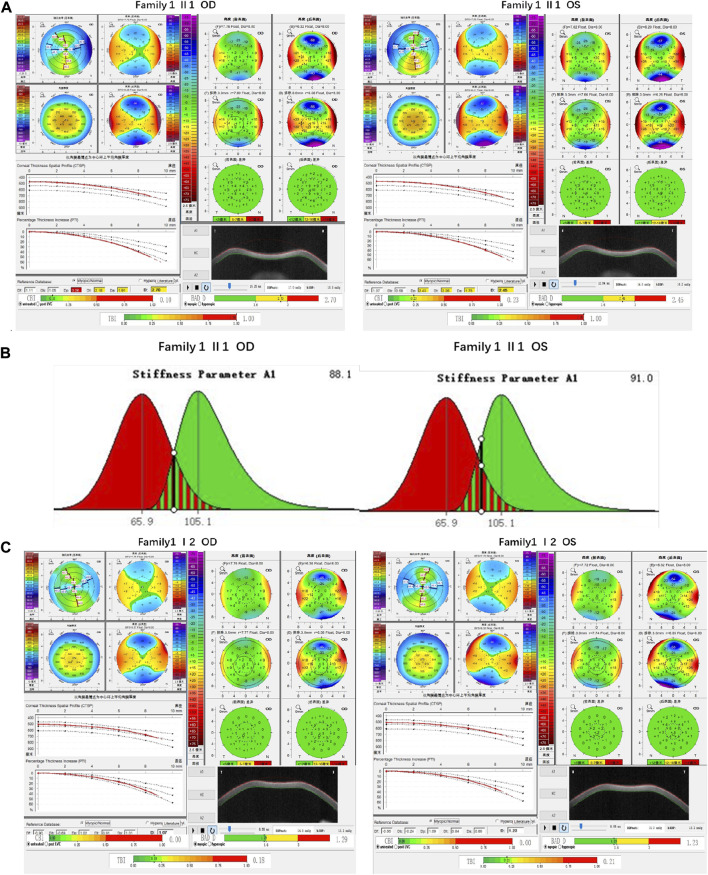
Corneal topographic map and corneal biomechanical examination in family 1. **(A)** TP, Dp (OS), Dt, Da, D, and BAD-D of probands are suspicious values (yellow); Dp (OD) and TBI are pathological values (red); PTI abnormalities: Showing abnormal distribution of corneal thickness; corneal thickness became thinner (471 μm in the right eye and 469 μm in the left eye); curvature: 45.3D below 43.0D above in the right eye, 47.1D below 43.0D above in the left eye (difference 4.1D below and above) **(B)** SP-A1<105.2 (88.1 in the right eye and 91 in the left eye): Smaller value, represent the greater the risk of corneal dilatation **(C)** All parameters of corneal topography and corneal biomechanics of the proband’s mother are normal.

**FIGURE 2:**
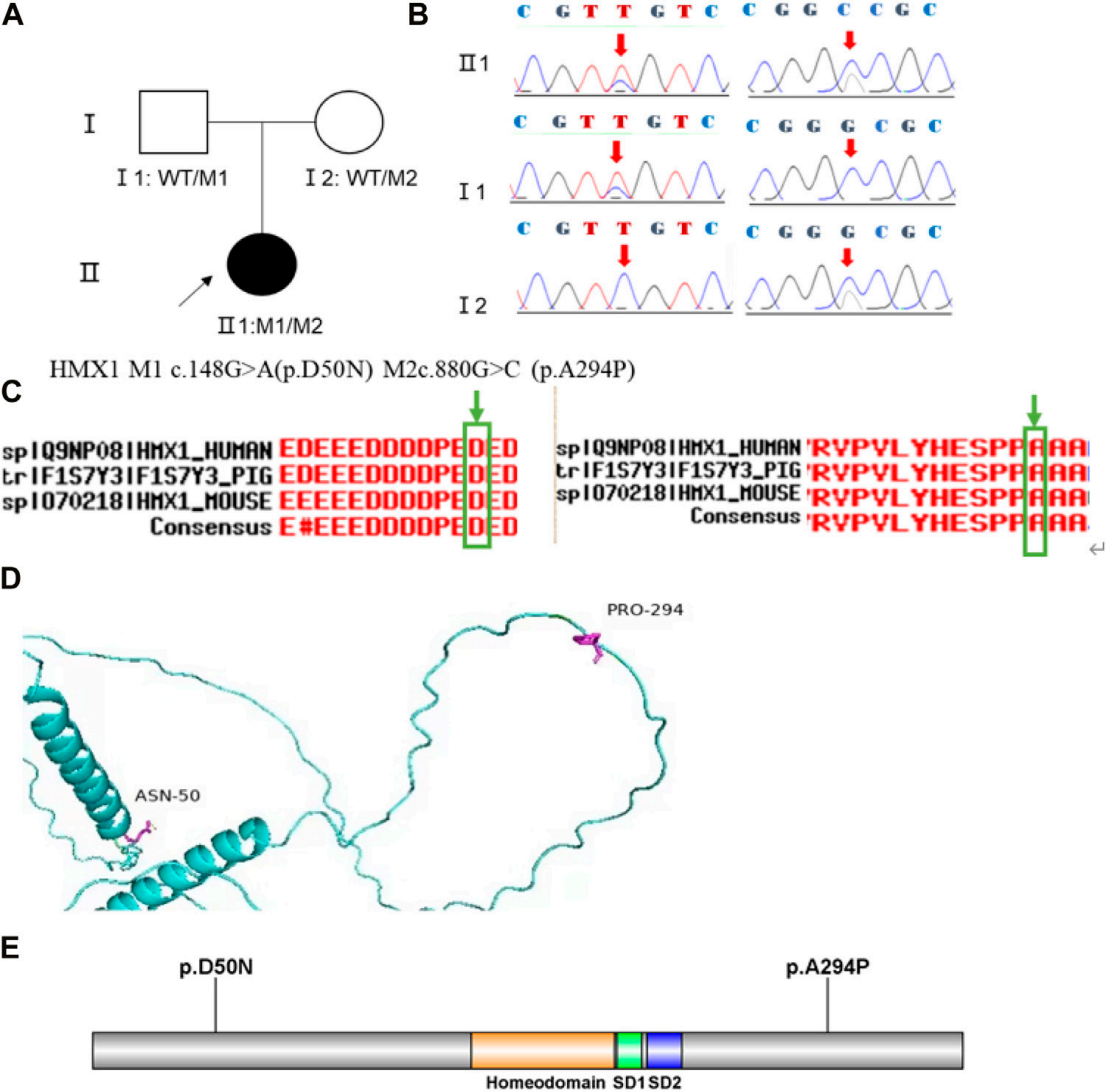
Sequence analysis and identification of the novel variants of HMX1 in the affected Chinese family with autosomal-recessive KC. **(A)** Pedigree of the family1. The filled black symbols represent the affected members, and the arrow denotes the proband. **(B)** By sequencing analysis, a compound heterozygous variant of **(C)**148 G > A and **(C)**880G > C were identified in the affected individual of II:1. **(C)** The homology of amino acid sequences between human HMX1 and other species. The amino acid at position 50 (Asparticacid, D50) and position 294(Alanine, A294) are highly conserved among species (green box). **(D)** Homology model of the HMX1 homeodomain (green). Purple indicates the location of p. Asp50Asn and p. Ala294Pro in the protein structure. **(E)** Structural modelling of HMX1 homeodomain.

Family 1 proband, 23-year-old female, best corrected visual acuity 1.0 (−5.00DS/−2.75DC × 180°) in the right eye and 1.0 (−6.00DS/−3.00DC × 180°) in the left eye, with thinning corneal thickness (471 μm in the right eye and 469 μm in the left eye) on Pentacam corneal topography. Corneal asymmetry in both eyes, right eye curvature: 43.0D above and 45.3D below, left eye curvature: 43.0D above and 47.1D below (4.1D difference between below and above). The PTI was abnormal in both eyes (Figure 1B), suggesting abnormal corneal thickness distribution. Belin analysis of several test indexes and monitoring indicators of corneal biomechanics showed suspicious or pathological values, and TBI was 1.0 in both eyes (Figure 1A). Slit lamp examination of the cornea did not show any significant abnormalities, and the diagnosis: was KC (incipient stage). Pentacam corneal topography, Belin analysis, and corneal biomechanics were performed on the proband’s parents, and no significant abnormalities were seen (Figure 1C). The compound heterozygous variants c.148G>A (p.Asp50Asn)and c.880G>C (p.Ala294Pro)were detected on the *HMX1* gene of the proband (Figure 2B), and the prediction of biological information suggested pathogenicity. As validated by Sanger, it was found that the proband’s father and mother with normal phenotype each carried a heterozygous missense variant (Figure 2), suggesting co-segregation of genotype and clinical phenotype. It was consistent with the autosomal recessive inheritance model and was finally diagnosed as autosomal recessive KC.

**FIGURE 3:**
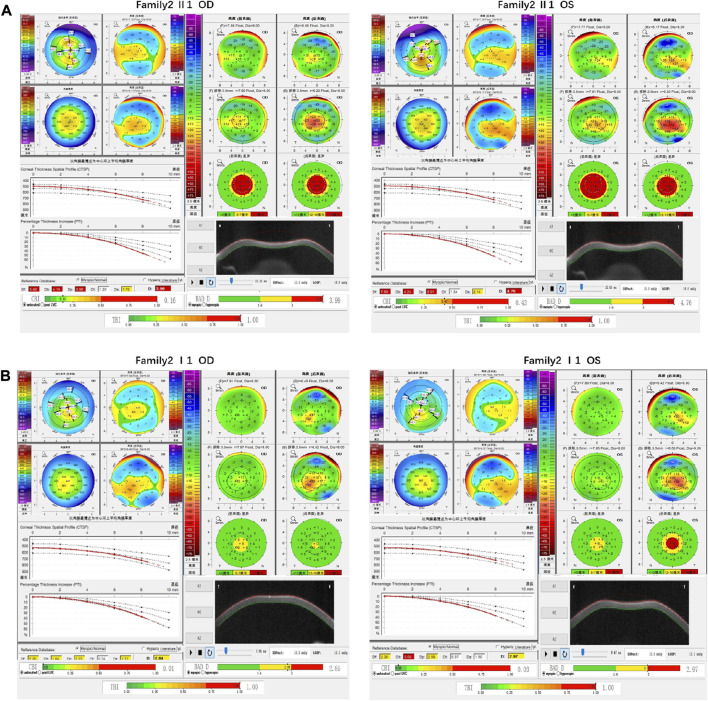
Corneal topographic map and corneal biomechanical examination in family 2. **(A)** For proband Ef, Eb, Df, Db, Dp, Da, D, BAD-D, CBI and TBI showing pathological values (red) and curvature: Kmax 47.6 D in the right eye and 48 D in the left eye **(B)** BAD-D, Df, Db, Dp, Dt, Da showing suspicious values (yellow) and TBI, Eb showing pathological values (red) in the father of proband.

**FIGURE 4:**
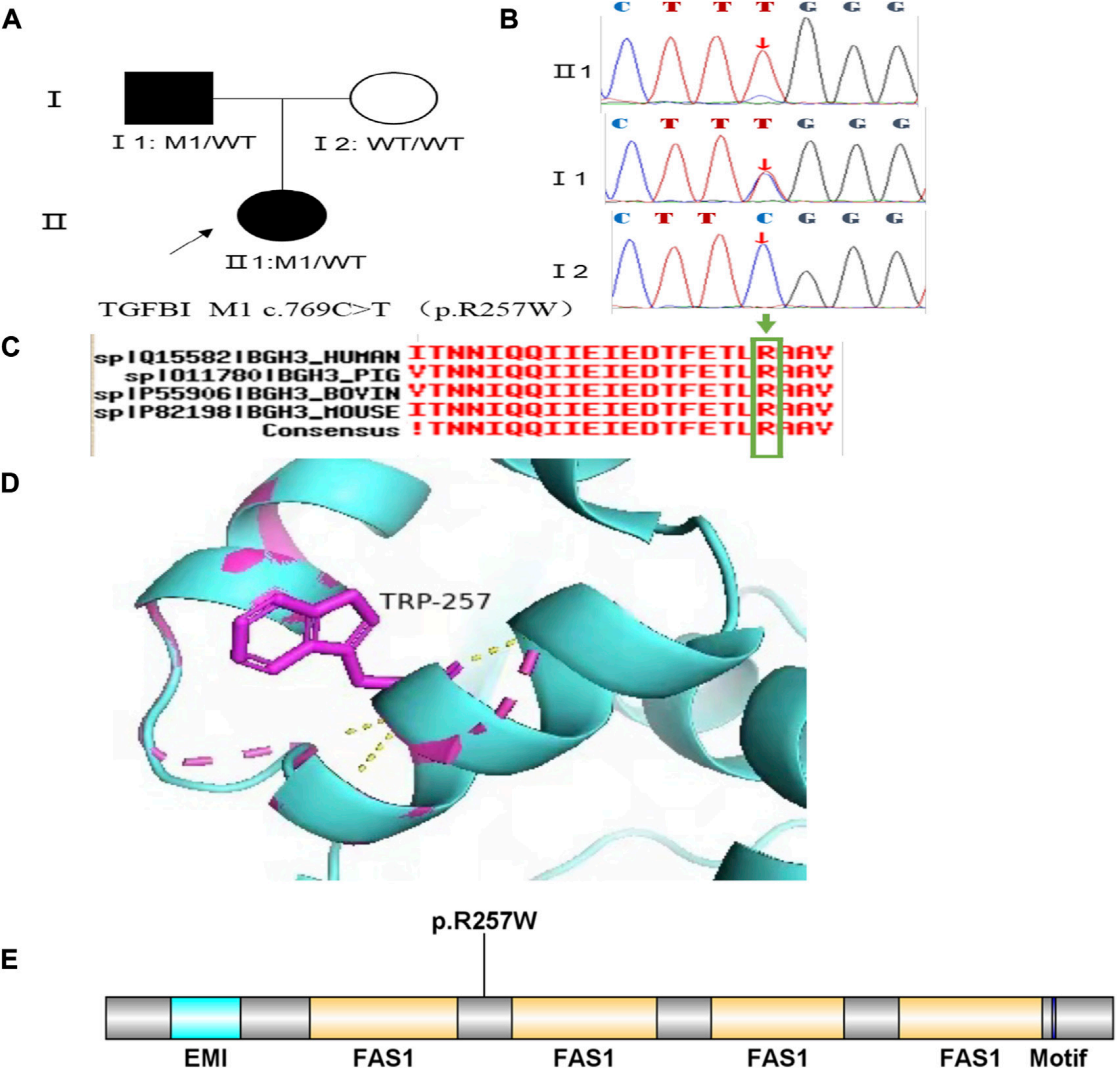
Sequence analysis and identification of the variant of TGFB1 in the affected Chinese family with autosomal-dominant KC. **(A)** Pedigree of the family. The filled black symbols represent the affected members, and the arrow denotes the proband. **(B)** By sequencing analysis, a heterozygous variant of **(C)**769 C > T was identified in the affected individuals of II:1 and I:1. **(C)** The homology of amino acid sequences between human TGFB1 and other species. The amino acid at position 257 (Arginine, R257) is highly conserved among species (green box). **(D)** Homology model of the TGF - β induced protein (green). Purple indicates the location of p. Arg257Trp in the protein structure. **(E)** Structural modelling of TGF - β induced protein homeodomain.

Family 2 proband, 9-year-old female, best corrected visual acuity 1.0 (−3.00DS/−1.25DC × 180°) in the right eye and 1.0 (−2.25DS/−1.75DC × 170°) in the left eye, steepened corneal curvature on Pentacam corneal topography, maximum anterior surface curvature 47.6D in the right eye and 48D in the left eye, posterior surface elevation value at the thinnest point of the cornea was greater than 13 μm in both eyes (14 μm in the right eye and 20 μm in the left eye). Belin analysis showed that all the test indexes were pathological values. Pentacam tomographic composite index (BAD-D) was pathological in both eyes (3.99 in the right eye and 4.76 in the left eye), and TBI was 1 in both eyes (Figure 3A). Slit lamp examination of the cornea showed no significant abnormalities, and the diagnosis: was KC (incipient stage). The father of the proband, 34 years old, had the best corrected visual acuity of 1.0 (−3.25 DS/−0.50DC × 25°) in the right eye and 1.0 (−2.75DS/−0.50DC × 120°) in the left eye. Pentacam corneal topography revealed a posterior surface elevation value greater than 16 μm at the thinnest point of the cornea in both eyes (18 μm in the right eye and 19 μm in the left eye). Belin analysis showed that all test indexes were suspicious values. The TBI was 1 in both eyes (Figure 3B). Slit lamp examination of the cornea showed no significant abnormalities. It was revealed that the father presented clinical characteristics of incipient KC. The heterozygous missense variant c.769C>T (p.Arg257Trp)was detected on the *TGFBI* gene of the proband (Figure 4B), and the prediction of biological information suggested pathogenicity. As validated by Sanger, the proband’s father carried the same heterozygous missense variant. The phenotypically normal mother was wild-type at this locus, suggesting co-segregation of genotype and clinical phenotype (Figure 4), consistent with the mode of an autosomal dominant inheritance.

**FIGURE 5:**
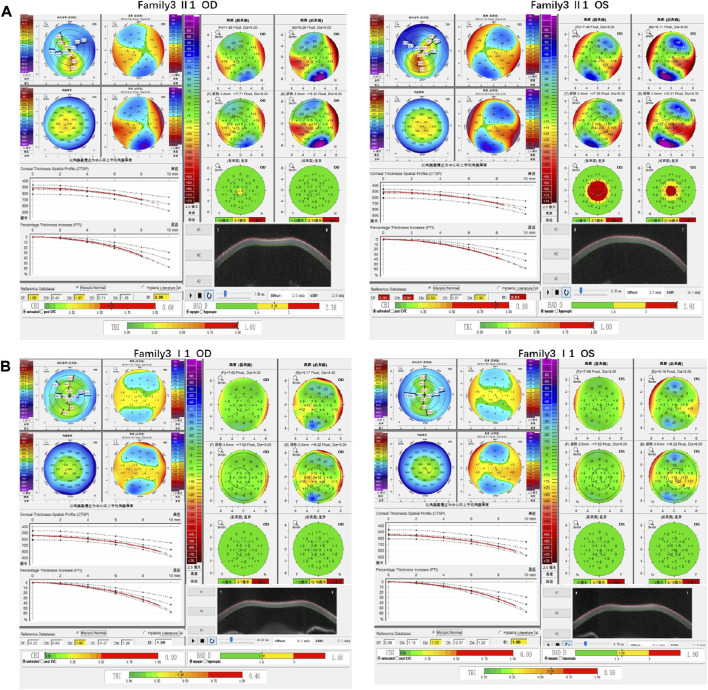
Corneal topographic map and corneal biomechanical examination in family3. **(A)** Kmax, Ef, Eb, Df, Db, Dp, Da, D, BAD-D, CBI and TBI showing pathological values (red) in the proband. **(B)** TDp, BAD-D, TBI is showing suspicious values (yellow) in the father of proband.

**FIGURE 6:**
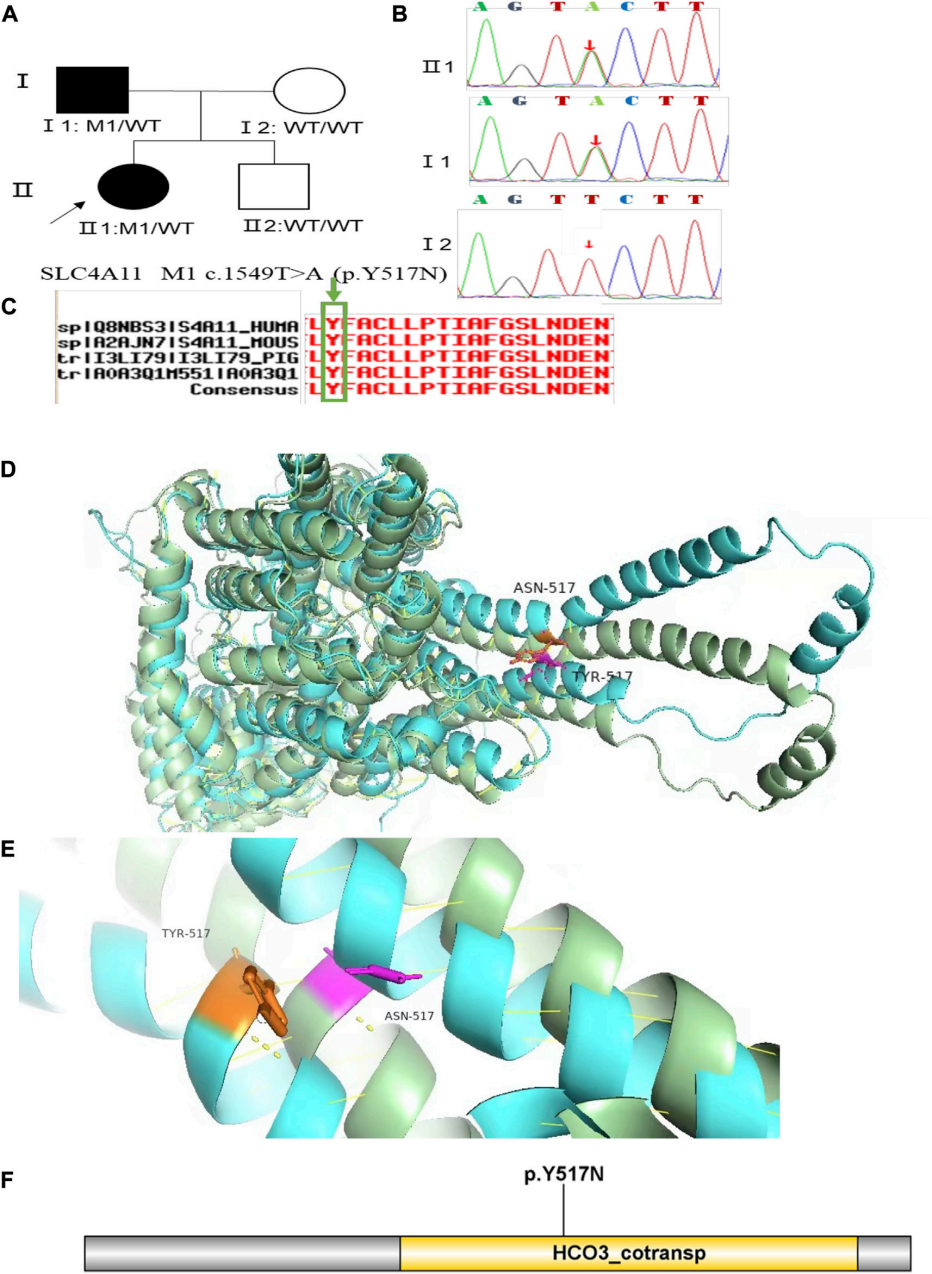
Sequence analysis and identification of the novel variant of SLC4A11 in the affected Chinese family with autosomal-dominant KC. **(A)** Pedigree of the family. The filled black symbols represent the affected members, and the arrow denotes the proband. **(B)** By sequencing analysis, a heterozygous variant of **(C)**1549T > A was identified in the affected individuals of II:1 and I:1. **(C)** The homology of amino acid sequences between human SLC4A11 and other species. The amino acid at position 517 (Tyrosine, Y517) is highly conserved among species (green box). **(D,E)** Homology model of the Solute carrier family 4 members 11 (green). **(F)** Structural modelling of Solute carrier family 4 members 11 homeodomain.

Family 3 proband, 23-year-old female, best corrected visual acuity 0.6 (−4.75DS/−2.75DC × 30°) in the right eye and 0.8 (−3.75DS/−4.50DC × 135°) in the left eye. Pentacam corneal topography revealed that the anterior and posterior surfaces of the cornea were abnormally elevated, with posterior surface elevation value greater than 13 μm at the thinnest point of the cornea (15 μm in the right eye and 21 μm in the left eye) and steepened corneal curvature, with a maximum anterior surface curvature of 47.9 D in the right eye and 50.3 D in the left eye. Belin analysis showed that several test indexes were pathological values. Pentacam tomographic composite index (BAD-D) was pathological in both eyes (2.38 in the right eye and 3.91 in the left eye), and TBI was 1 in both eyes (Figure 5A). Diagnosis: KC (completion stage I). The father of the proband, 47 years old, did not show any significant abnormalities on slit lamp examination. Belin analysis showed that the corneal thickness progression deviation (Dp) value in both eyes was in the suspicious value state (1.96 in the right and 1.73 in the left eye). The TBI was suspicious in both eyes (0.46 on the right and 0.59 on the left) (Figure 5B). The heterozygous missense variant c.1549T>A (p.Tyr517Asn)was detected on the *SLC4A11* gene of the proband (Figure 6B), and the prediction of biological information suggested pathogenicity. As validated by Sanger, the proband’s father carried a heterozygous variant at the same locus. The phenotypically normal mother was wild-type at this locus, suggesting co-segregation of genotype and clinical phenotype (Figure 6), consistent with the mode of an autosomal dominant inheritance.

**FIGURE 7:**
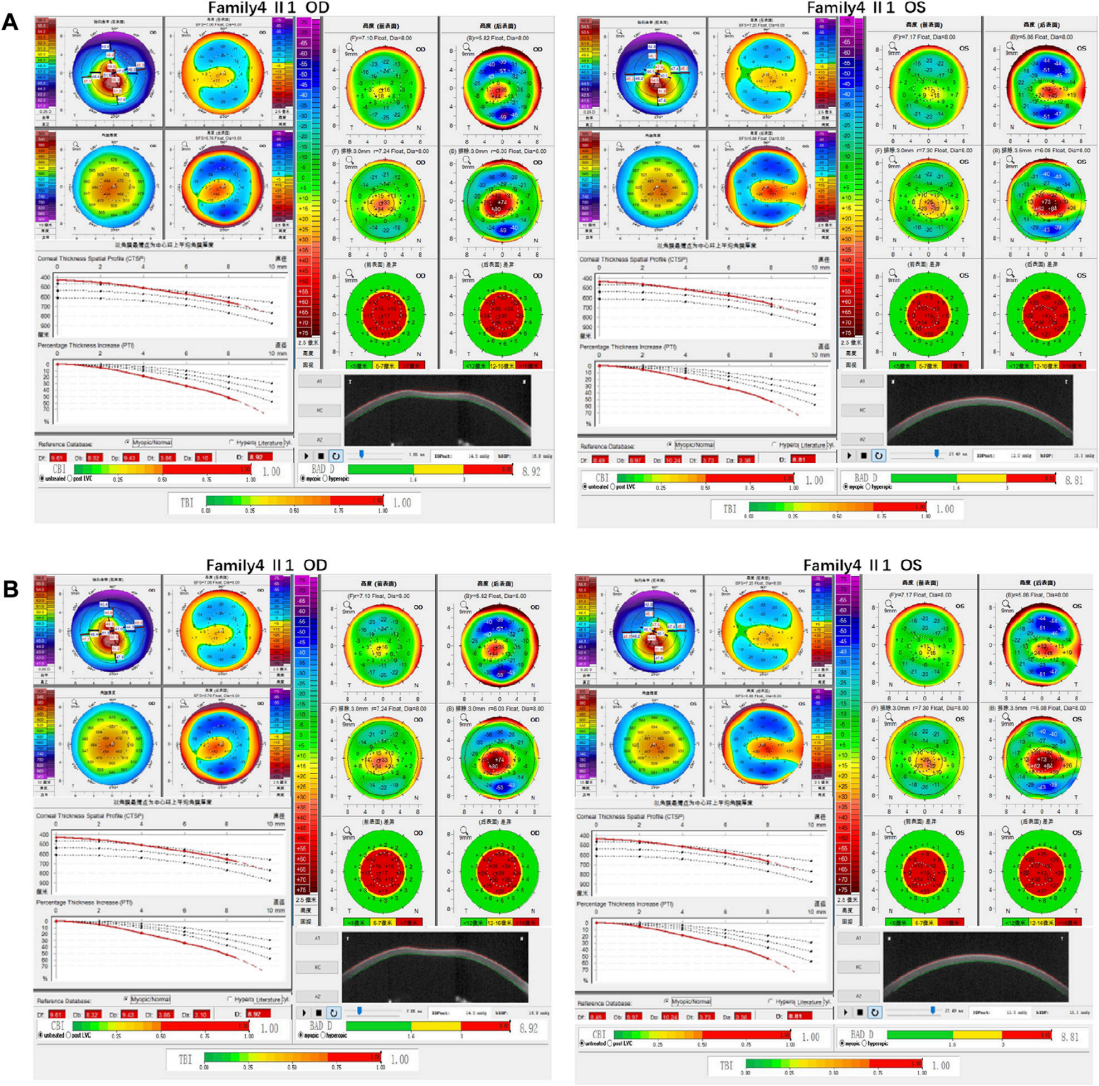
Corneal topographic map and corneal biomechanical examination in family4. **(A)** Kmax, TP, Ef, Eb, Df, Db, Dp, Dt, Da, D, BAD-D, CBI and TBI showing pathological values (red) in the proband. **(B)** Kmax, Eb, Df, Db, Dp, Dt, Da, BAD-D, TBI showing suspicious values (yellow). CBI and TBI are pathological values (red) in the father of the proband.

**FIGURE 8:**
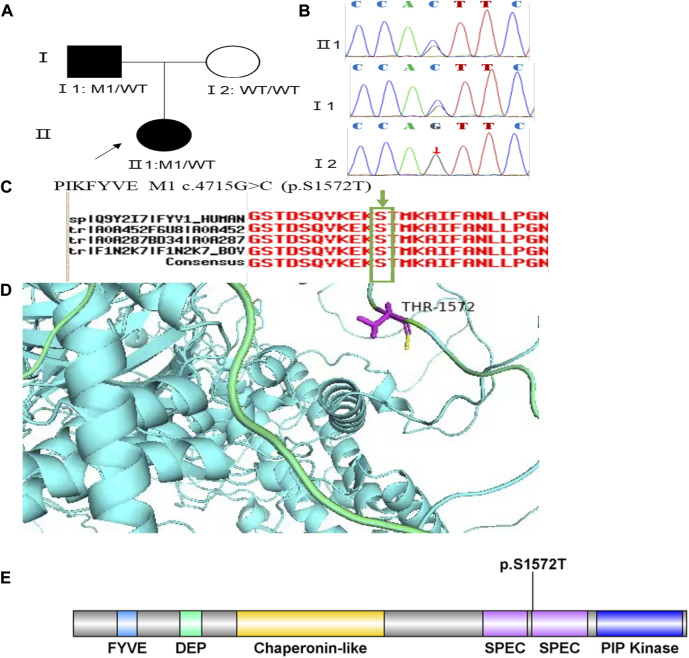
Sequence analysis and identification of the novel variant of PIKFYVE in the affected Chinese family with autosomal-dominant KC. **(A)** Pedigree of the family. The filled black symbols represent the affected members, and the arrow denotes the proband. **(B)** By sequencing analysis, a heterozygous variant of **(C)**4715G > C was identified in the affected individuals of II:1 and I:1. **(C)** The homology of amino acid sequences between human PIKFYVE and other species. The amino acid at position 1,572 (Serine, S1572) is highly conserved among species (green box). **(D)** Homology model of the 1-phosphatidylinositol 3-phosphate 5-kinase (green). Purple indicates the location of p. Ser1572Thr in the protein structure. **(E)** Structural modelling of 1-phosphatidylinositol 3-phosphate 5-kinase homeodomain.

Family 4 proband, 25-year-old female, best corrected visual acuity 0.4 (−2.50DS/−3.50DC × 25°) in the right eye and 0.6 (−1.50DS/−2.75DC × 150°) in the left eye. Pentacam corneal topography revealed that the anterior and posterior surfaces of the cornea were abnormally elevated, with anterior surface elevation value (Ef) greater than 11 μm at the thinnest point in both eyes (20 μm in the right eye and 17 μm in the left eye), posterior surface elevation value (Eb) greater than 16 μm at the thinnest point in the cornea (46 μm in the right eye and 47 μm in the left eye), steepened corneal curvature, maximum anterior surface curvature (58.8 D in the right eye and 55.6 D in the left eye), and thinning corneal thickness (430 μm in the right eye and 433 μm in the left eye). Belin analysis showed that all the test indexes were pathological values, and the CBI and TBI were 1.0 in both eyes (Figure 7A). Diagnosis: KC (completion stage I). The father of the proband, 46 years old, showed no significant abnormalities on slit lamp examination. Pentacam corneal topography revealed that the corneal curvature became steeper, the maximum curvature of the anterior surface was 48 D in the right eye and 48.4D in the left eye, and the corneal thickness became thinner (446 μm in the right eye and 460 μm in the left eye). Belin analysis showed that all the test indexes were pathological values; the TBI was close to 1 in both eyes (0.94 in the right and 0.99 in the left eye) (Figure 7B). It was shown that the father presented clinical characteristics of incipient KC. The heterozygous mutation c.4715G>C (p.Ser1572Thr) was detected on the *PIKFYVE* gene of the proband (Figure 8B), and the prediction of biological information suggested pathogenicity. As validated by Sanger, the proband’s father carried a heterozygous variant at the same locus. The phenotypically normal mother was wild-type at this locus, suggesting co-segregation of genotype and clinical phenotype (Figure 8), consistent with the mode of an autosomal dominant inheritance.

**FIGURE 9:**
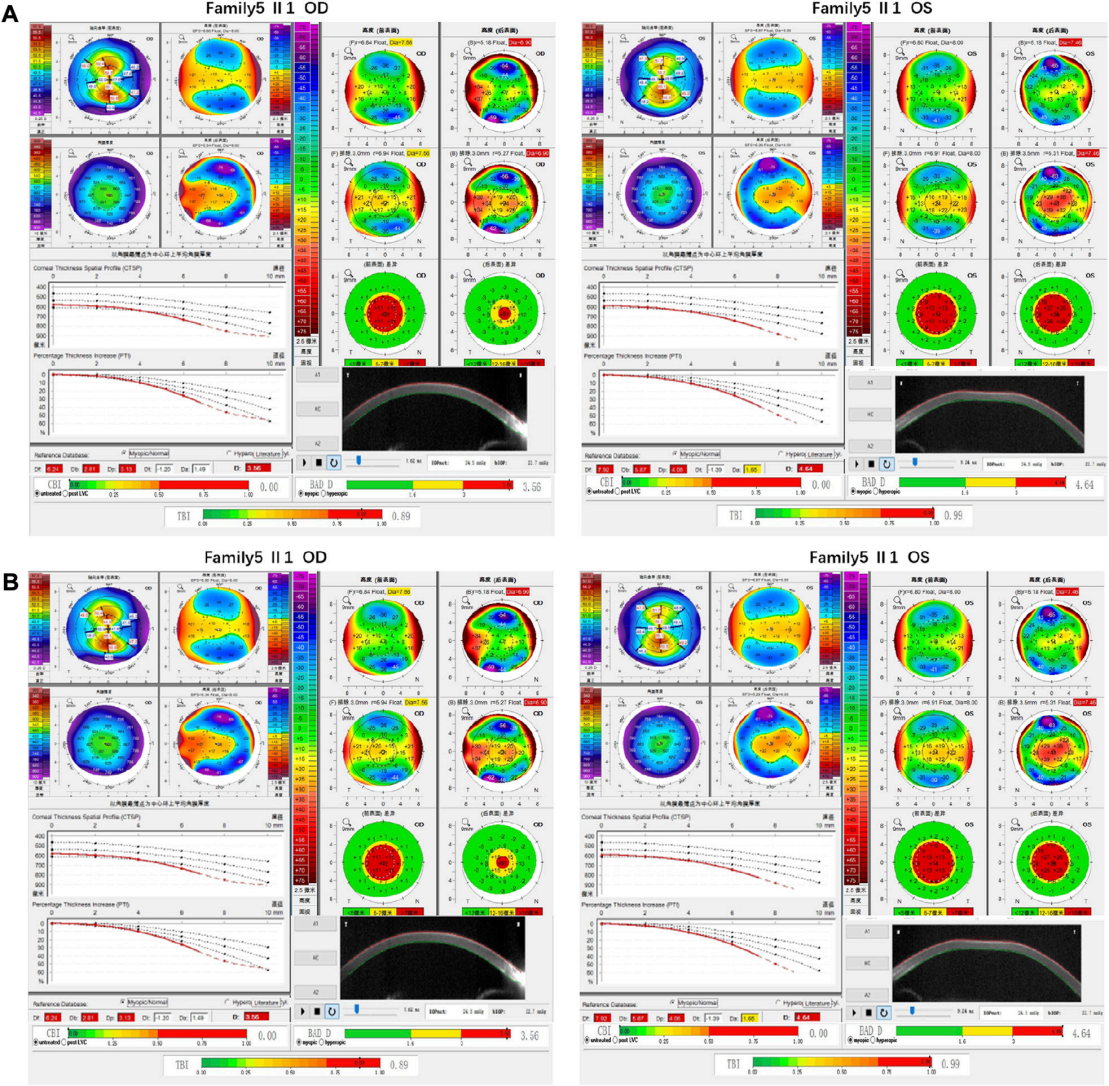
Corneal topographic map and corneal biomechanical examination in family5. **(A)** Kmax, Ef, Df, Db, Dp, D, BAD-D, TBI showing pathological values (red) in the proband. **(B)** BAD-D showing suspicious values (yellow) and Kmax, TBI showing pathological values (red) in the proband’s mother.

**FIGURE 10:**
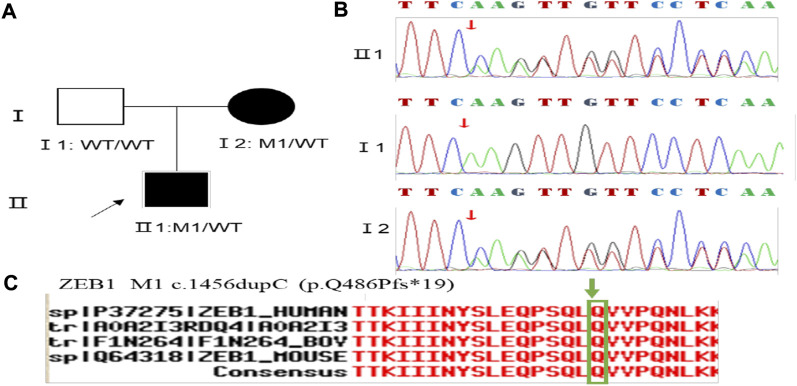
Sequence analysis and identification of the novel variant of ZEB1 in the affected Chinese family with autosomal-dominant KC. **(A)** Pedigree of the family. The filled black symbols represent the affected members, and the arrow denotes the proband. **(B)** By sequencing analysis, a heterozygous variant of **(C)**1456 dup C was identified in the affected individuals of II:1 and I:1. **(C)** The homology of amino acid sequences between human ZEB1 and other species. The amino acid at position 486 (Glutamine, Q486) is highly conserved among species (green box).

Family 5 proband, 8-year-old male, best corrected visual acuity 0.6 (−1.00S/−5.00DC×5°) in the right eye and 0.6 (−1.00DS/−3.50DC×10°) in the left eye. Pentacam corneal topography revealed that the corneal curvature became steeper and the maximum curvature of the anterior surface (55.2D in the right look and 55.5D in the left eye). The anterior surface elevation value (Ef) at the thinnest point of the cornea was more significant than 8 μm in both eyes (9 μm in the right eye and 10 μm in the left eye). Belin analysis showed that all the test indexes were pathological values. Pentacam tomographic composite index (BAD-D) was pathological in both eyes (3.56 in the right eye and 4.64 in the left eye), and TBI was close to 1 in both eyes (0.89 in the right eye and 0.99 in the left eye) (Figure 9A). Diagnosis: KC (Completion stage I). The mother of the proband 32 years old. Pentacam corneal topography revealed that the corneal curvature became steeper, the maximum curvature of the anterior surface (50.5D in the right eye and 51.2D in the left eye). The posterior surface elevation value (Eb) at the thinnest point in the right eye’s cornea was 19 μm. Belin analysis showed that all the test indexes were pathological values; the TBI was close to 1 in both eyes (0.87 in the right and 0.75 in the left eye) (Figure 9B). It was shown that the mother presented clinical characteristics of incipient KC. The heterozygous frameshift variant c.1456dupC (p.Gln486Profs*19)was detected on the *ZEB1* gene of the proband (Figure 10B), and the prediction of biological information suggested pathogenicity. In the family verification analysis, the proband’s mother carried a heterozygous variant at the same locus. The phenotypically normal father was wild-type at this locus, suggesting co-segregation of genotype and clinical phenotype (Figure 10), consistent with the mode of an autosomal dominant inheritance.

### 3.2 Results of bioinformatics analysis

In family 1, the novel compound heterozygous missense variants c.148G>A (p.Asp50Asn) and c.880G>C (p.Ala294Pro) (PM5_Supporting) were initially detected in exon one and exon two of the *HMX1* gene of the proband (Ⅱ:1) by NGS. They then segregated the disease status, it was later confirmed that a missense variant c.148G>A from the unaffected father (Ⅰ:1) and the second missense variant c.880G>C from the natural mother (Ⅰ:2) by Sanger sequencing (PP1_Supporting). These two variants have not been previously reported in the literature and were also not detected in the ordinary people database of gene sequencing companies (Agilent Technologies, Inc.). The search for the variants on the five HGMD Professional Databases (1,000 genomes, ESP6500, Inhouse, ExAC_ALL, and ExAC_EAS) showed a very low frequency of these two variants in the control population (Table1) (PM2_Supporting). Therefore, the pathogenicity of the two novel *HMX1* variants was further predicted by subsequent analysis of the function of the relevant protein. The first variant (c.148G>A), the nucleotide at position 148 in the coding region being variant from G (guanine) to A (adenine), would cause the amino acid change from Asparticacid into Asparagine at residue 50 (p.Asp50Asn). The second variant (c.880G>C), the nucleotide at position 880 in the coding region being variant from G (guanine) to C (cytosine), would cause the amino acid change from Alanine to Proline at residue 294 (p.Ala294Pro). In the prediction of protein function of the two variants, three software, PolyPhen-2 (score: 0.97; probably damaging), SIFT (score:0.045; deleterious), and Mutation Taster (score:1), suggested the harmful impact of the two substitutions (Table 1). Moreover, the Asparticacid at position 50 and Alanine at position 249 were highly conserved among different species by proteomic conservation analysis (Figure 2C), indicating that the two variants at these sites are more likely to affect the structure and function of HMX1 protein (PP3_Supporting). The p. Asp50Asn variant resides in the Poly-Asp COMPBIAS (Figure 2D), leading to the Poly-Asp COMPBIAS being lost, downstream of the altered splice site might get lost. The p. Ala294Pro variant resides in the Pro-rich region (Figure 2D), leading to the Pro-rich region being lost, downstream of the altered splice site might get lost. Which is a DNA-binding protein that binds to the 5′-CAAG-3′ core sequence. It may function as a transcriptional repressor. Seems to act as a transcriptional antagonist of NKX2-5. May play an important role in the development of craniofacial structures such as the eye and ear. Missense 3D predicts that the substitution at 294 positions would trigger a clash alert. The local clash score for wild type is 7.30 and the local clash score for mutant is 35.46, meanwhile triggering a disallowed phi/psi alert. The phi/psi angles are in the favored region for wild-type residue but the outlier region for mutant residue. We, therefore, believed that the compound heterozygous variants c.148G>A (p.Asp50Asn) and c.880G>C (p.Ala294Pro) of the *HMX1* gene were more likely the pathogenic variants of family 1 with KC according to ACMG guideline.

**TABLE 1:** Pathogenicity analyses of variants and bioinformatics analysis of variants in the 5 Chinese families with KC.

	Family1	Family2	Family3	Family4	Family5
Gene	HMX1	TGFBI	SLC4A11	PIKFYVE	ZEB1
Nucleotide	c.148G>A	c.769C>T	c.1549T>A	c.4715G>C	c.1456dupC
	c.880G>C				
Amino acid	p.Asp50Asn p.Ala294Pro	p.Arg257Trp	p.Tyr517Asn	p.Ser1572Thr	p.Gln486Profs*19
Chromosome	chr4:8,873,193	chr5:135,383,107	chr20:3,210,411	chr2:209,204,184	chr10:31,809,718–31,809,718
	chr4:8,869,586				
Exon	exon1	exon6	exon13	exon30	exon7
	exon2				
Source	father and mother	father	father	father	mother
1000g2015aug_all	-	-	-	-	-
ESP6500s	-	-	-	-	-
Inhouse		0.00022			
ExAC_ALL	-	0.00001658	-	-	-
ExAC_EAS	-	0.0001	-	-	-
SIFT	0.045	0.001	0.817	0.143	NA
	0.335				
PolyPhen_2	0.053	0.97	0.969	0.927	NA
	0				
Mutation Taster	11	1	1	1	NA
GERP++	0.794	5.13	3.7	4.21	NA
	1.6				

In family 2, the heterozygous missense variant c.769C>T (p.Arg257Trp) was detected in exon 6 of the *TGFBI* gene in the proband (Ⅱ: 2) by NGS and then segregated the disease status, it was later confirmed that the same variant was detected in proband’ affected father (I:1) and unaffected mother (I:2) was wild-type at this locus by Sanger sequencing (PP1_Supporting). The incidence of the variant in the population was extremely low, showed a very low frequency in the 1000g2015aug_all, ESP6500si databases, and 0.00022, 0.00001658, and 0.0001 for Inhouse, ExAC_ALL, and ExAC_EAS, respectively (Table 1) (PM2_Supporting). Therefore, the pathogenicity of the variant c.769C>T (p.Arg257Trp was further predicted by subsequent analysis of the function of the relevant protein. The nucleotide at position 769 in the coding region was variant from C (cytosine) to T (thymine), and the substitution would cause the amino acid change from Arginine to Glutamine at residue 257 (p.Arg257Trp). In the prediction of protein function of the variant, three software, PolyPhen-2 (score: 0.97; probably damaging) SIFT (score:0.001; damaging), and Mutation Taster (score: 1), suggested the deleterious impact of this substitution. What’s more, the Arginine at position 257 was highly conserved among different species by proteomic conservation analysis with GERP++ score >2 (Figure 4C; Table 1), indicating that the variant at this site is more likely to affect the structure and function of TGFBI protein (PP3_Supporting). The p. Arg257Trp variant resides in the FAS1-2 domain (Figure 4D), leading to the FAS1-2 domain being lost. Human TGFBI is a multi-domain 683-residue protein, which contains one CROPT domain and four FAS1 domains. The FAS1 domains are sandwiches of two orthogonal four-stranded β sheets decorated with two three-helix insertions. The overall TGFBIp architecture discloses regions for integrin binding and that most dystrophic mutations cluster at both molecule ends (Garcia-Castellanos et al., 2017). We, therefore, believed that the heterozygous missense variant c.769C>T (p.Arg257Trp) of the *TGFBI* gene was more likely the pathogenic variant of family 2 with KC according to ACMG guidelines.

In family 3, the heterozygous missense variant c.1549T>A (p.Tyr517Asn) was detected in exon 13 of the *SLC4A11* gene in the proband (Ⅱ: 2) by NGS and then segregated the disease status; it was later confirmed that the same variant was detected in proband’ affected father (I:1) and unaffected mother (I:2) was wild-type at this locus by Sanger sequencing (PP1_Supporting). The incidence of this variant was extremely low in the population, and a search for the variants on the five HGMD Professional Databases (1,000 genomes, ESP6500, Inhouse, ExAC_ALL, and ExAC_EAS) showed a very low frequency of these two variants in the control population (Table 1) (PM2_Supporting). Therefore, the pathogenicity of the variant c.1549T>A (p.Tyr517Asn) was further predicted by subsequent analysis of the function of the relevant protein. The nucleotide at position 1,549 in the coding region was variant from T (Thymine) to A (Adenosine), and the substitution would cause the amino acid change from Tyrosine to Asparagine at residue 517 (p.Tyr517Asn). In the prediction of protein function of the variant, two software, PolyPhen-2 (score: 0.969; probably damaging) and Mutation Taster (score:1), suggested the deleterious impact of this substitution (Table 1). What’s more, the Tyrosine at position 517 was highly conserved among different species by proteomic conservation analysis with GERP++ score >2 (Figure 6C; Table 1), indicating that the variant at this site is more likely to affect the structure and function of SLC4A11 protein (PP3_Supporting). The p. Tyr517Asn variant resides in the Membrane (bicarbonate transporter) region (Figure 6D), leading to the Membrane (bicarbonate transporter) region being lost. Mammalian bicarbonate transport proteins are involved in a wide range of physiological processes. Bicarbonate is the waste product of mitochondrial respiration. Defects of bicarbonate transport proteins manifest in diseases of most organ systems (Cordat and Casey, 2009). We, therefore, believed that the heterozygous missense variant c.1549T>A (p.Tyr517Asn) of the *SLC4A11* gene was more likely the pathogenic variant of family 3 with KC according to ACMG guidelines.

In family 4, the heterozygous missense variant c.4715G>C (p.Ser1572Thr) was detected in exon 30 of the *PIKFYVE* gene in the proband (Ⅱ: 2) by NGS and then segregated the disease status, it was later confirmed that the same variant was detected in proband’ affected father (I:1) and unaffected mother (I:2) was wild-type at this locus by Sanger sequencing (PP1_Supporting). The incidence of this variant was extremely low in the population, and a search for the variants on the five HGMD Professional Databases (1,000 genomes, ESP6500, Inhouse, ExAC_ALL, and ExAC_EAS) showed a very low frequency of these two variants in the control population (Table 1) (PM2_Supporting). Therefore, the pathogenicity of the variant c.4715G>C (p.Ser1572Thr) was further predicted by subsequent analysis of the function of the relevant protein. The nucleotide at position 4,715 in the coding region was variant from G (guanine) to C (cytosine), and the substitution would cause the amino acid change from Serine to Threonine at residue 1,572 (p.Ser1572Thr). In the prediction of protein function of the variant, two software, PolyPhen-2 (score: 0.927; probably damaging) and Mutation Taster (score:1), suggested the deleterious impact of this substitution (Table 1). What’s more, the Serine at position 1,572 was highly conserved among different species by proteomic conservation analysis with GERP++ score >2 (Figure 8C, Table 1), indicating that the variant at this site is more likely to affect the structure and function of PIKFYVE protein (PP3_Supporting). The p. Ser1572Thr variant resides in the Phosphoserine MOD_RES (Figure 8D), leading to the Phosphoserine MOD_RES being lost. Phosphorylation of proteins on serine and threonine residues has traditionally been viewed as a means to allosterically regulate catalytic activity. It can also directly result in the formation of multimolecular signaling complexes (Yaffe and Elia, 2001). We, therefore, believed that the heterozygous missense variant c.4715G>C (p.Ser1572Thr) of the *PIKFYVE* gene was more likely the pathogenic variant of family 4 with KC according to ACMG guidelines.

In family 5, the heterozygous frameshift variant c.1456dupC (p.Gln486Profs*19) was detected in exon 7 of the *ZEB1* gene in the proband (Ⅱ: 2) by NGS. It then segregated the disease status, and it was later confirmed that the same variant was detected in the proband’ affected mother (I:2) and unaffected father (I:1) was wild-type at this locus by Sanger sequencing (PP1_Supporting). The search for the variants on the five HGMD Professional Databases (1,000 genomes, ESP6500, Inhouse, ExAC_ALL, and ExAC_EAS) showed a shallow frequency of these two variants in the control population (Table 1) (PM2_Supporting). Nucleotide C (cytosine) was duplicated at position 1,456 in the coding region, and the variant resulted in an amino acid change from Glutamine to Proline at residue 486, followed by an early stop codon after frameshift of 19 amino acids (p.Gln486Profs*19) (PP3_supporting). A nonsense variant can lead to no transcription products or transcriptional degradation, resulting in complete deletion of gene products and destruction of gene function (PVS1_Very Strong). We, therefore, believed that the frameshift variant c.1456dupC (p.Gln486Profs*19) of the *ZEB1* gene was more likely the pathogenic variant of family 5 with KC according to ACMG guidelines.

To summarize, each of the six pathogenic variants is most likely the causative mutation for the disease phenotype in the corresponding family (Table 1). This is because: 1) The six variants were observed in the database of normal subjects of the sequencing company with a shallow variant frequency or were no detections; 2) There are no other potential pathogenic variants detected in this study; 3) There is a well-established correlation between the gene variant and the disease phenotypes for each family.

## 4 Discussion

With advances in molecular genetic science in recent years, there is increasing evidence that KC is associated with genetic factors. Approximately 11–14% of relatives without obvious clinical symptoms may be found to have suspicious KC if a detailed ophthalmologic examination, including slit lamp examination, corrected visual acuity, and corneal topography. Corneal biomechanics is performed on the first-degree relatives of KC patients (Steele et al., 2010). A study by Rabinowitz et al. (Rabinowitz et al., 2020) on Arabian, KC patients showed that consanguineous marriages might result in a significantly higher KC incidence, with a family history of the disease in about 5–23% of cases, which support the idea that genetic factors play a crucial influence in the development of KC. As studied by (Levy et al., 2004)^,^ suspicious corneal morphological abnormalities were found in at least one eye after detailed ophthalmic examination in KC relatives carrying the relevant genetic variant loci but without obvious clinical symptoms. Matthew and Charles (2001) 90% of familial KC exhibited autosomal dominant inheritance, and other inheritance modes had also been described in some literature, including autosomal recessive and X-linked inheritance.

The *HMX1* gene (H6 family homeobox frame 1, MIM #612109) is located at 4p16.1, encoding a homeodomain transcription factor mainly restricted to sensory organs, gill (gill) pharyngeal arches, and the central nervous system. It is expressed at an early stage of embryonic development and is required to develop sensory organs (Gillespie et al., 2015a). Ocular development is controlled by a complex mechanism consisting of transcription factors, cell cycle regulators, etc. *HMX1* gene variants may affect such tools and thus lead to restricted ocular development. Homozygous variants within *HMX1* cause Oculoauricular syndrome, an autosomal recessive disorder (MIM: 612,109). A study by (Gillespie et al., 2015b) described a consanguineous family originating from Switzerland, a homozygous deletion variant in the first exon of the *HMX1* gene, that leads to Oculoauricular syndrome characterized by microcornea, microphthalmia, anterior-segment dysgenesis, cataract, coloboma of various parts of the eye, and a particular cleft ear lobule. Boisset and Schorderet (2012) identified a homozygous missense variant within the highly conserved homeodomain of the *HMX1* gene that leads to complex developmental ocular abnormalities of congenital cataract, anterior segment dysgenesis, iris coloboma, early-onset retinal dystrophy, and abnormal external ear cartilage presented in the affected family members. *HMX1*gene appears to play an important role in the development of the anterior segment, axonal guidance, and the establishment of polarity within the retina (Schorderet et al., 2008; Gillespie et al., 2015b). KC is a genetic disorder characterized by abnormal corneal development, accompanied by anterior segment abnormalities such as microcornea. It is usually not associated with ear abnormalities. Previously, it was reported that repression of the *HMX1* gene in zebrafish resulted in microphthalmia and anterior segment hypoplasia (Boisset and Schorderet, 2012). In this study, the novel compound heterozygous missense variants c.148G>A (p.Asp50Asn) and c.880G>C (p.Ala294Pro) were detected in the *HMX1* gene in the proband of family 1 with KC. The proband of family 1 presented solely ocular phenotypes and did not show recognizable signs of other systemic organs. And the phenotype is mild and the main manifestation is KC. *HMX1* variants have been associated with both syndromic and non-syndromic ocular dysplasia, presumably due to whether the variants affect single or multiple transcripts, or to the heterogeneity of clinical phenotypes. Thus, it can be inferred that the compound variants of c.148G>A and c.880G>C in the *HMX1* gene are responsible for autosomal -recessive KC pathogenesis in family 1.

The *TGFBI* gene (TGF - β induced protein, MIM #601692) is located at 5q31.1, which is an extracellular protein that mediates cell adhesion to laminin, fibronectin, etc. Is involved in maintaining corneal epithelial-stromal structure and function. Transforming growth factor (TGF) family members play important roles in the regulation of corneal integrity, and the pathogenesis of corneal fibrosis (Chen et al., 2019)^.^ In 1997 the first set of DNA variants was identified in the transforming growth factor beta-induced (*TGFBI*) gene for anterior corneal dystrophies (Munier et al., 1997). The *TGFBI* corneal dystrophy is implicated in autosomal dominant disorder Corneal dystrophy on OMIM. *TGFBI* gene is expressed by both the corneal epithelial cells and the keratocytes, showing phenotypic heterogeneity. KC is a noninflammatory, bilateral, progressive corneal stromal thinning disorder, central anterior corneal stromal scarring, irregular myopic astigmatism, and reduced visual acuity. Corneal dystrophy is bilateral, sporadic, autosomal dominant, and non-inflammatory. As the dystrophy evolves, the major complaints include glare, halos, and reduced visual acuity (Gurnani et al., 2021). Very rarely, both the pathologies can co-exist in the same patient (Lin et al., 2022). Reported a case of a Chinese Han family with hereditary KC in which a novel variant c.1406G > A in the *TGFBI* gene was found by genetic testing, demonstrating the association of this gene with the development of KC disease. In this study, it can be inferred that the variant of c.769C>T (p.Arg257Trp) in the *TGFBI* gene is responsible for autosomal-dominant KC pathogenesis in family 2.

The *SLC4A11* gene (MIM #610206) is located at 20p13, encoding a hydrophilic membrane protein involved in intra- and extracellular carbon dioxide transport and regulation of intracellular pH, and genetic variants can lead to corneal dystrophy and perceptive deafness (Harboyan syndrome), as well as dominant late-onset Fuchs endothelial corneal dystrophy (FECD) (Romero et al., 2004). Two blinding corneal dystrophies, pediatric-onset congenital hereditary endothelial dystrophy (CHED), and some cases of late-onset Fuchs endothelial corneal dystrophy (FECD) are caused by *SLC4A11* variants (Malhotra et al., 2019). CHED is a rare autosomal recessive disorder of the corneal endothelium characterized by nonprogressive bilateral corneal edema and opacification present at birth, (Patel and Parker, 2015). Reviewed that CHED individuals have variants in *SLC4A11* which encodes a transmembrane protein in the SLC4 family of bicarbonate transporters. In the study of candidate genes related to KC disease, (Karolak et al., 2020) eventually identified a total of 35 genes with differential expression in KC compared to non-KC patients, of which two genes, *TGFBI* and *SLC4A11*, had the most pronounced differences in expression and were also included as candidate genes for KC pathogenesis. In family 3, the proband and her father carried the same heterozygous variant of the *SLC4A11* gene, which was pathogenic after bioinformatic analysis and conservativeness analysis.

The *PIKFYVE* gene (MIM #609414) is located at 2q34, is involved in the phosphatidylinositol signaling pathway, and participates in building the lipid structure function of cell membranes, which is essential to maintaining normal membrane fluidity and function (Li et al., 2005; Boisset et al., 2008). Boisset et al. (2008) by cloning the *PIKFYVE* gene, found that such gene contains 42 exons and is widely expressed in adult human organs, localized in specific cell types such as the cornea, lens, ganglion cell layer, and inner nuclear layer. Li et al. (2005) reported that human *PIKFYVE* gene variants were associated with speckled corneal dystrophy. In family 4, the proband and her father carried the same heterozygous variant of the *PIKFYVE* gene.

The *ZEB1* gene (zinc finger Ebox binding homeobox one gene, MIM #189909), located at 10p11.22, is the gene encoding a zinc finger transcription factor that is closely associated with eye development. Mazzotta et al. (2014) reported a case of a patient with KC, Epithelial Basement Membrane Dystrophy (EBMD), and Fuchs corneal endothelial dystrophy carrying variants in the *ZEB1* gene. Chung et al. (2019) found that alterations in the *ZEB1-OVOL2-GRHL2* gene axis (caused by mutations in PPCD-related genes) resulted in corneal endothelial cell morphology and molecular alterations pathways, including abnormal activation of the Wnt signaling pathway. Zhao et al. (2021) reported a KC family with 2 KC patients, the proband and his father, in the family. Whole-exome sequencing revealed that the proband and his father carried heterozygous mutations in the *ZEB1* gene (c.643G>C, p. V215L). The study showed that the *ZEB1* variant was responsible for the development of KC in this family. This was also the first time the *ZEB1* variants were identified in a simple KC. In family five under this study, the proband and the mother carried the same heterozygous variant of the *ZEB1* gene, genetic testing combined with the clinical phenotype indicated that the heterozygous variant was pathogenic after bioinformatic and conservativeness analysis.

This study used genomic DNA target sequence capture combined with high-throughput sequencing to identify genetic variant loci associated with KC or corneal degeneration, corneal dystrophy, and other corneal diseases in all five families. The pathogenicity of the variant loci was analyzed accordingly. Meanwhile, Sanger sequencing combined with clinical phenotyping was also applied to validate the family co-segregation. The results showed that the loci found in all five families were potentially pathogenic.

With the development of corneal topography and anterior segment analysis techniques and the availability of some powerful analytical tools, significant progress has been made in studying the heritability of corneal traits in KC patients. Genetic factors are essential in features such as corneal curvature, thickness, and posterior surface elevation. Li et al. (2020) used the Pentacam corneal topography and the Belin system to analyze the corneal traits of the parents of KC patients. They found that the parents of patients had early occult changes of subclinical KC. The corneal thickness of the parents of KC patients was thinner than that of the healthy population, with an increase in posterior corneal surface elevation of about 9 μm, which had specific characteristics of subclinical KC. Wang et al. (2010) by comparing the central corneal curvature (CK), the asymmetry index (I-S value) of the upper and lower corneal refractive power, and the corneal irregularity and astigmatism index (KISA) between the first-degree KC relatives and normal controls, found that these KC-related qualitative indices showed the heredity. It was demonstrated that these indicators could be used as predictors for early detection of KC with quantifiability. In this study, a more precise assessment of corneal morphology by corneal topography combined with Belin analysis and corneal biomechanical examination revealed that all relatives of KC patients carrying the same pathogenic gene variant locus had abnormal corneal morphology indicators, proving the existence of a genetic predisposition to corneal morphological abnormalities. Due to the different penetrance of genetic variants, some first-degree KC relatives, although carrying genetic variants, also have other clinical manifestations and severity. The clinical phenotype of the first-degree relatives may be atypical, and vision is often not seriously affected. Therefore, detecting abnormalities without a detailed corneal morphological examination is difficult.

As the most important contraindication for refractive surgery, accurate preoperative screening and diagnosis of KC are particularly critical. However, the early clinical manifestations of KC are not obvious, and the overlap and low sensitivity of clinically specific indicators often happen in some keratoconus and some normal corneas with abnormal morphology or thin thickness, which has been a difficulty for ophthalmologists in the early diagnosis of keratoconus. (Belin et al., 2022). Previous studies have shown that the occurrence of KC is closely related to genetic factors. Notwithstanding, as a highly congenital heterogeneous disease, the pathogenesis of KC is still unclear. Still using NGS technology has confirmed that some variants of nuclear genes are responsible for keratoconus. In recent years, with the advance in genetic research, genetic testing technology has been clinically applied to the diagnosis of genetic ocular diseases. Numerous clinical studies have shown that genetic testing is helpful in the early diagnosis and prevention of the disease. In addition, screening for pathogenic variants provides value for the molecular genetic pathogenesis of KC. Establishing the genetic research’s relationship between genotype and phenotype is an important task.

## 5 Conclusion

This study provides evidence that variants in *HMX1, SLC4A11, TGFBI, PIKFYVE,* and *ZEB1* genes were associated with KC, which variants in these five genes are known to be responsible for KC or corneal degeneration, corneal dystrophy, and other corneal diseases according to previous studies. The clinical phenotype of autosomal dominant KC showed irregular dominant inheritance, in which different individuals in the same family carried the same pathogenic heterozygous variants. Still, the severity of corneal morphological changes was different. It was also found that some characteristic of corneal morphology is hereditary, and a detailed ophthalmic examination of the first-degree relatives of KC patients may help in early screening of the disease. Our study also extends the gene spectrum associated with KC, provides novel insights into KC phenotypic assessments, and contributes to early diagnosis and treatment for these patients. The deficiency of our study is that the number of clinical families included is not large enough to perform a holistic analysis of the disease. In subsequent studies on KC, it is necessary to collect many KC families for a comprehensive examination to clarify the characteristics of the pathogenic genes and corneal morphological inheritance associated with KC.

## Data Availability

The datasets presented in this study can be found in online repositories. The names of the repository and accession number can be found below: Banklt repository (BankIt (https://www.ncbi.nlm.nih.gov/WebSub/) ID: 2627340)
